# Effects of Adapted Physical Activity Programs on Body Composition and Sports Performance in a Patient with Parkinson’s Disease: A Case Report

**DOI:** 10.3390/healthcare13243195

**Published:** 2025-12-05

**Authors:** Luciana Zaccagni, Natascia Rinaldo, Gaetano Campanale, Antonio Pastore, Francesca Rametta, Emanuela Gualdi-Russo

**Affiliations:** 1 Department of Neuroscience and Rehabilitation, Faculty of Medicine, Pharmacy and Prevention, University of Ferrara, Corso Ercole I d’Este 32, 44121 Ferrara, Italy; luciana.zaccagni@unife.it (L.Z.);; 2 Associazione Parkinsoniani & Caregiver, Piazza Buozzi 14/A, Pontelagoscuro, 44123 Ferrara, Italy; g.campanale87@gmail.com (G.C.); f.rametta@airis.it (F.R.); 3 Piscina Bacchelli, Via R. Bacchelli 103, 44121 Ferrara, Italy

**Keywords:** Parkinson’s disease, adapted swimming, exercise, body composition, sports performance

## Abstract

The benefits of physical activity on physical and mental health are well established. Exercise can be particularly advantageous in neurodegenerative disorders such as Parkinson’s disease (PD), where progressive loss of muscle mass and impaired motor performance are common. We report the case of a 58-year-old man with PD who underwent a structured, adapted physical activity program in preparation for a relay swim across the Strait of Messina (Sicily, Italy). The aim was to evaluate changes in body composition (fat mass, fat-free mass) and performance following four months of adapted swimming training, alongside adapted physical activity in the gym and Nordic walking. The patient swam 1300 m in 42 min and 38 s in the relay, which was a marked improvement from the baseline and subsequent assessments. In conclusion, while a longer follow-up period and a larger patient sample would be necessary, the findings from this case study suggest that the adapted exercise program improved both physical fitness and body composition. This generally supports the key role of physical activity in managing Parkinson’s disease and, in particular, the positive effects of adaptive sports training.

## 1. Introduction

Parkinson’s disease (PD), a complex disorder influenced by both genetic and environmental factors (pesticides, herbicides, heavy metals), is one of the most prevalent neurodegenerative conditions. Motor symptoms typically include the triad of tremor, rigidity, and bradykinesia, with postural instability developing as the disease progresses [1].

Physical activity has consistently been shown to benefit individuals across all age groups. In PD specifically, exercise may enhance motor learning and behavioral performance by promoting neuroplasticity and restoring basal ganglia circuits [2]. Aerobic and resistance training, in particular, have been recommended in recent guidelines as integral components of PD management [3,4,5,6]. Despite this evidence, heterogeneity in training protocols and outcome measures often limits direct comparison among studies and prevents the identification of optimal exercise modalities [7]. Weight dysregulation—ranging from undernutrition to excess adiposity—is also common in PD and represents a critical yet underrecognized concern [8]. Many patients exhibit increased fat mass coupled with lean mass depletion, both of which reduce motor efficiency, functional independence, and quality of life [8,9]. For this reason, the assessment of body composition provides a sensitive indicator of training effectiveness in PD rehabilitation.

Moreover, sex-related biological and physiological differences may influence both disease expression and exercise response. Women with PD often experience more pronounced fatigue and postural instability, whereas men tend to present with greater rigidity and strength-related adaptations [10,11]. Even though the present case report involves a male participant, acknowledging these dimensions supports a more nuanced interpretation of training effects.

Although numerous studies have examined the effects of aquatic or adapted physical activity (APA) in patients with Parkinson’s disease, most have focused on short-term, clinic-based outcomes rather than real-world performance contexts. As highlighted in recent reviews [12,13,14], there remains a lack of longitudinal, ecologically valid case studies exploring how structured APA protocols might influence both functional performance and body composition. Considering that PD patients frequently exhibit an unfavorable balance between fat and lean mass, our study focused on these parameters as key indicators of physical adaptation.

Therefore, the present case report, structured in accordance with the CARE guidelines [15], aimed to evaluate the effects of a combined APA program—encompassing gym-based exercise, Nordic walking, and adapted swimming—on body composition and sports performance in a male PD patient preparing for “Swim for Parkinson,” a charity relay event across the Strait of Messina, promoted by the Limpe Foundation for Parkinson’s Onlus under the patronage of the Italian Swimming Federation and the Italian Paralympic Swimming Federation.

We hypothesized that a structured, individualized APA program would (i) improve anthropometric and performance parameters, particularly by reducing fat mass while preserving lean mass and (ii) demonstrate the feasibility and motivational value of competitive, adapted aquatic training in PD management.

## 2. Materials and Methods

### 2.1. Participant and Program Overview

The study participant was a 58-year-old man with a 4-year history of PD (in Hoehn and Yahr Stage 2), followed by neurologists and physiatrists at the University Hospital of Ferrara, Italy. Based on his prior active lifestyle (including work as a martial arts instructor), technical ability, and strong motivation, he was deemed eligible by the medical team to participate in the 2022 Swim for Parkinson initiative. The inclusion criteria were as follows: Hoehn and Yahr Stage ≤ 2; a 12-min swim test > 200 m; and completion of the training program. The relay event consisted of a 3.5 km swim across the Strait of Messina, connecting Sicily and mainland Italy.

A CARE-compliant timeline, summarizing all major milestones from the initial diagnosis of PD to the final event, is reported in Table 1.

### 2.2. Intervention

Table 2 summarizes the adapted physical activity (APA) program, reporting the frequency, intensity (heart rate), time, adherence, and type of tasks for each training modality.

In preparation, the participant trained in both the pool and the gym. Following 18 months of APA in the gym and Nordic walking, which he maintained throughout the study, he completed a four-month adapted swimming program in a 25 m pool in the four months leading up to crossing the Strait. The gym-based APA sessions focused on dual-tasking, gait autonomy, trunk control, coordination, and endurance through functional re-education exercises, Nordic walking, and group sessions involving music-stimulated movement. The adapted swimming sessions included a 10–15 min warm-up, 25–30 min of technical drills, and a 5–10 min cool-down. Once the participant’s aerobic capacity and stroke mechanics improved, the central training expanded to include drills incorporating stroke variations, style transitions, and speed changes. In preparation for the competition, over the last month, he trained once a week in the open sea.

The details of the APA program, according to the FITT (frequency, intensity, time, and type of exercise) principles, are described in the Supplementary File (Supplementary S1). In particular, swimming training intensity was monitored using heart rate, which should not exceed 150 beats per minute. Maintaining HR below 150 bpm ensured that the athlete stayed within a moderate aerobic zone, favoring predominant oxidative metabolism, improving endurance efficiency, and reducing cardiovascular strain during training.

A replicable protocol was proposed in the Supplementary File (Supplementary S2).

All swimming sessions were supervised by a certified kinesiologist (P.A.) and took place in a therapeutic pool with a water temperature of around 28 °C. The participant trained alone in the lane, wearing a swimming cap, swimsuit, and goggles. He was always accompanied in the water, and some sessions took place in the shallow area of the pool, where the water temperature was approximately 32 °C, which is known to be optimal for muscle relaxation, soothing muscle fatigue, and relieving body tension. The activities were specifically designed to accommodate the coordination and balance limitations typical of PD. Rest periods were provided as needed to avoid excessive fatigue.

The safety criteria for open-water swimming included a stable clinical status, an absence of acute symptoms, adherence to prescribed medication, the use of a brightly colored swimming cap, wetsuit, and goggles, under the supervision of qualified personnel, who followed him in a kayak, and in favorable sea conditions. Progression in training was only applied when the participant completed all planned sessions without adverse signs or excessive fatigue. Conversely, load reduction or regression was implemented in the presence of unusual dyspnea, musculoskeletal discomfort, or environmental risk factors.

These criteria were used to adjust both pool-based and open-water sessions throughout the program. No adverse events were reported during the intervention. In particular, during the race, which took place in the morning, with calm seas and no waves and a water temperature of around 25 °C, each participant had a support boat with a caregiver and a doctor on board, in addition to the sailor, and safety buoys and safety jackets.

The prescribed medication regimen consisted of Ropinirole 8 mg once a day/Madopar 100 + 25 mg four times a day/Opicapone 50 mg once a day. No formal pharmacological and dietary monitoring was undertaken during the intervention; instead, the participant, in a condition of pharmacological stability, was advised to maintain his usual diet throughout the study period.

Motor performance was assessed at baseline, periodically during the swimming program, and again during the relay event.

### 2.3. Anthropometric and Performance Assessments

The subject arrived at the Anthropometry Laboratory of the University of Ferrara in the morning after fasting. The hydration status was stable, no vigorous exercise had been performed in the preceding 12 h, and the subject was adherent to the prescribed medication regimen. The environmental temperature was maintained within an appropriate range (23 °C during the first assessment and 24 °C during the second).

Standard anthropometric protocols were followed for somatometric and physiometric measurements to ensure reproducibility [16,17]. Somatometric traits included standing height, weight, waist circumference (WC), mid-upper-arm circumference (MUAC) in both relaxed and contracted states, and skinfold thickness at the triceps, biceps, subscapular, and suprailiac sites. In particular, standing height was measured to the nearest 0.1 cm using an anthropometer (GPM, Zurich, Switzerland), and body weight was measured to the nearest 0.1 kg using a calibrated electronic scale. WC was measured to the nearest 0.1 cm using a non-stretchable tape measure at the midpoint between the iliac crest and the lowest rib. MUAC was measured to the nearest 0.1 cm using a non-stretchable tape measure at the midpoint between the acromion and the olecranon process of the left elbow, in two positions: with the arm relaxed by the side and the elbow straight (MUAC relaxed), and with the arm raised, the elbow bent, the fist closed and the muscle contracted (MUAC contracted) [17,18]. Skinfold thicknesses were measured to the nearest 0.5 mm using a Lange caliper (Beta Technology Inc., Houston, TX, USA) on the left side [19]. All anthropometric traits were measured by the same trained anthropometrist (L.Z.), whose TEMs (assessed before the project) were <5% for skinfolds and <1% for other measurements.

Derived indices included BMI, waist-to-height ratio (WHtR), and arm fat index (AFI). BMI was calculated by dividing the weight (in kilograms) by the height squared (in meters). This index was used to assess the weight status of the participant according to WHO cut-off values (2000) [20]. WHtR was calculated by WC/height [21]. The upper arm fat index (AFI) was obtained using Frisancho’s equations [18], based on relaxed mid-upper arm circumference (MUAC) and triceps skinfold measurements.

Due to the lack of validated body composition equations specifically developed for PD patients, body fat percentage (%F) was estimated using skinfold measurements according to the Durnin and Womersley equations for body density [22], followed by conversion through Siri’s formula [23], and subsequent calculations of fat mass (FM) and fat-free mass (FFM) in kilograms, in addition to Fat Mass Index (FMI = FM/height^2^) and Fat-Free Mass Index (FFMI = FFM/height^2^) calculated in kg/m^2^. The Durnin and Womersley equations are widely used in clinical and field-based assessments. While they may not provide highly accurate absolute estimates of adiposity, they are considered reliable for tracking relative changes in individuals over time, which was the primary aim of this study.

Physiometric measures included spirometry—vital capacity (VC), forced vital capacity (FVC), and forced expiratory volume in one second (FEV1)—by a COSMED T170 spirometer (Torino, Italy), handgrip strength measured using a Takei 5001 GRIP-A handgrip dynamometer (Takei, Tokyo, Japan), and the Digit Symbol Substitution Test (DSST) performed to reveal potential improvements in attention, processing speed, memory, and executive functioning.

The swimming training program was designed based on results from the 12-Minute Swim Test, an adapted version of the Cooper test for swimming [24]. In this test, the participant attempts to cover the greatest possible distance in 12 min. The program also considered the participant’s stroke rate over 25 m, which is the number of strokes required to swim this distance. Metrics recorded during training with a Garmin Swim™ 2 (Garmin Ltd., Milan, Italy) included average stroke rate, swimming pace, heart rate, and SWOLF score. SWOLF (Swim Golf) score is an efficiency index commonly used in swimming: it combines the time needed to cover a given distance with the number of strokes taken. A lower SWOLF score generally indicates greater efficiency, meaning the swimmer can swim the same distance with fewer strokes and/or in less time. Although it is not a clinical metric in a strict sense, it is often used as a practical indicator of technical economy and swim performance.

## 3. Results

**Figure 1:**
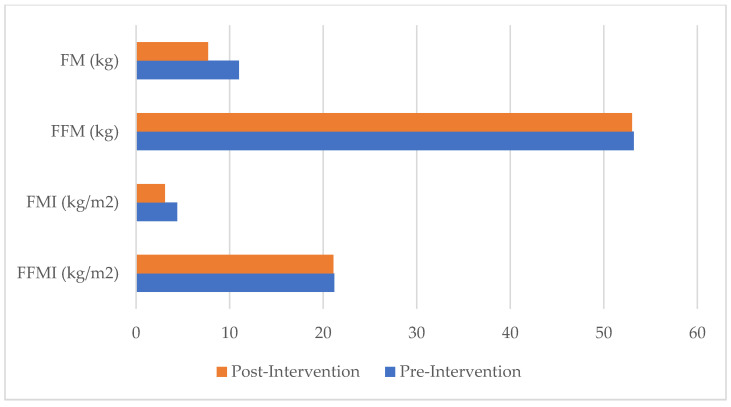
Changes in body composition parameters between pre- and post-intervention.

Table 3 and Figure 1 show the changes in the patient’s anthropometric traits and body composition before and after participation in the swimming program. Unlike other anthropometric traits, height was measured only once, at the initial assessment.

Changes in somatometric traits were observed when pre- and post-intervention data were compared, with clear decreases in weight (5.5%), waist circumference (5.9%), WHtR (5.6%), skinfold thicknesses (25% at the biceps; 11.1% at the triceps; 27.3% at the subscapular site; 30.8% at the suprailiac site), the sum of these (24.3%), and relaxed MUAC (4.4%). Consequently, all parameters indicating body adiposity changed considerably: BMI decreased by 5.5%, %F by 4.4%, FM and FMI by 30%, and AFI by 6.6%. At the same time, there were few to no significant changes recorded in the muscular component: FFM and FFMI decreased by 0.4%, and MUAC (contracted) remained unchanged. The FFMI value is in the 90th percentile for both repetitions, while the FMI value is at the 25th percentile for the first measurement and moves to the 10th percentile for the second, according to the reference values by sex and age proposed by Schutz et al. [25] based on a large sample of Caucasian subjects.

Table 3 also reports the physiometric changes detected at the end of the APA program. Spirometric parameters, whose values were within the normal range [26], showed an increase in VC (8.2%) and FEV1 (1.2%), while FVC decreased by 2.6%. Handgrip strength increased more markedly in the left hand (+10%) than in the right (+3.2%). The DSST score also increased by 21.1% compared to the initial value.

**Figure 2:**
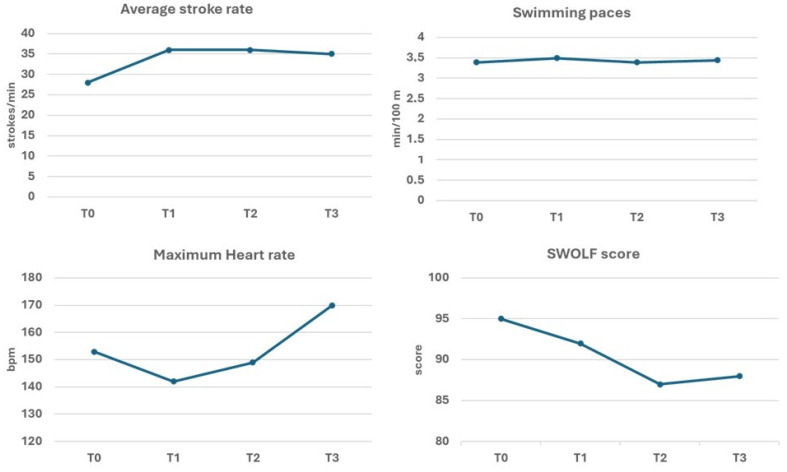
Changes in motor test performance from baseline values (T0) over four months of swimming training (T1 and T2) and in the final relay (T3).

Figure 2 shows the results of the motor tests performed repeatedly in the pool (at the beginning, T0, and on two subsequent occasions) and during the swim across the Strait of Messina (T3). Compared to the baseline, all measured parameters showed an improvement trend. In particular, the average stroke rate per minute increased by 29%, while the swimming pace over 100 m showed only minor fluctuations. The maximum heart rate decreased considerably in T1 (−7%) as a result of the training effect. In T2, it increased but remained below both the initial value and the threshold of 150 bpm. The maximum HR reached its highest value during the race, exceeding even the baseline measurement. The SWOLF score decreased progressively until it reached 8.4% of the initial score at the second repetition (T2), and a similar score was maintained in the final competition.

The Swim for Parkinson’s program saw this participant increase his swimming distance from 650 m to 1300 m, a feat achieved in a time of 42′38″ with a total of 1588 strokes. This accomplishment was made possible by preparatory work in the gym, pool, and open sea.

## 4. Discussion

This case report documents how exercise was employed not only for rehabilitation purposes but also to enhance performance, enabling the patient to reach his full potential and optimize his competitive outcomes in relay swimming across the Strait of Messina. Considering that the literature on PD patients mainly concerns cycling, dance, and Nordic walking outside of sporting contexts [27,28,29,30], the study of motor adaptation in swimming for a relay race shows some innovative aspects. The intervention yielded significant improvements in anthropometric, physiometric, and motor performance parameters. Although these findings cannot be generalized, this study aims to document the case systematically, providing therapists and coaches with essential, reproducible information and stimulating future research in specific real-world settings.

From a body composition standpoint, the participant shifted from being slightly overweight to a normal weight, primarily due to a reduction in fat mass while preserving lean mass. In particular, the results of this study demonstrate that the weight loss observed at the end of the training program can be attributed to changes in body composition, due to a significant reduction in body fat (%F, FM, FMI, skinfold thicknesses, AFI). Conversely, the muscle component, as indicated by FFM, FFMI, and contracted MUAC, showed minimal variation during this period. Given that PD is commonly associated with muscle loss and excess adiposity [8], this stability in FFM is a particularly encouraging outcome. It should be noted that, in the absence of PD-specific formulas, body composition parameters were derived using Durnin and Womersley’s equations. Although originally validated in healthy individuals, these methods are widely used in clinical research for monitoring relative changes over time. For this reason, body composition parameters should primarily be interpreted in terms of relative changes, rather than absolute accuracy, even in this case study. Integrating instrument-based methods, such as bioelectrical impedance analysis (BIA) and dual-energy X-ray absorptiometry (DXA), may improve accuracy and capture disease-specific body composition features. Finally, the WC and WHtR decreased during this period, reaching values close to those considered acceptable according to WHO standards (2008) [31].

Physiometric and cognitive improvements were observed, particularly in respiratory function, handgrip strength, and DSST performance. The concurrent gains in DSST performance could also be partially related to the dual-task elements embedded in the gym training sessions. Dual-task training—which requires simultaneous cognitive processing and motor execution—has been shown to enhance processing speed, attention shifting, and executive functioning in PD, as demonstrated in both randomized controlled trials and meta-analyses [32,33]. These domains are central to DSST performance, which relies on rapid symbol–number associations under time pressure. While causality cannot be inferred in a single case, the integration of cognitive–motor challenges during land-based exercise may have contributed to the observed improvement. Grip strength, which at the initial assessment was already above the mean value reported for Italian men aged 55–59 in the Longevity Check-Up (Lookup) 7+ project [34], increased further, particularly in the left hand, reaching the 75th percentile.

Regarding the interpretability of the functional improvements, it must be acknowledged that no minimal clinically important difference (MCID) has been formally established for DSST in PD. Nevertheless, psychometric studies in neurologically healthy adults and validation studies of digital DSST versions report good test–retest reliability (ICC ≈ 0.82–0.88) and modest practice effects (typically 1–2 points) [35,36]. Therefore, although this remains a single-case observation, the +4-point improvement shown by our participant is likely to exceed expected measurement variability. Handgrip strength likewise shows excellent reliability in older adults and in PD cohorts (ICC > 0.95), with standard errors of measurement and minimal detectable changes commonly reported around 1–2 kg [37,38]; therefore, the participant’s improvements (+3.2% right; +10% left) appear to exceed typical measurement error, although interpretation should remain cautious and framed within the exploratory nature of a single-case design. The participant showed improvements in swimming time and stroke efficiency. The improvement in SWOLF observed in this case indicates enhanced stroke efficiency. A more efficient stroke pattern typically corresponds to better swimming economy, reducing the oxygen cost per unit distance and lowering cardiovascular load during submaximal swimming. This is particularly relevant for individuals with PD, who often show increased metabolic cost of movement and reduced mechanical efficiency. Therefore, the participant’s improved SWOLF may reflect a more economical and sustainable swimming technique. More generally, improved swimming metrics indicated enhanced aerobic and anaerobic capacities, as well as greater stroke efficiency, particularly in the breaststroke. This proved to be the safest and most comfortable style for the patient. Despite the increased difficulties due to environmental factors such as swimming in open water, these results were also evident in the final swim: he swam a distance more than twice as far as he could initially during the relay crossing of the strait. These findings align with evidence suggesting that higher-intensity exercise may induce greater benefits in PD, including neuromuscular adaptations [39], and are consistent with recent meta-analyses on aquatic exercise (“hydrotherapy”) in Parkinson’s disease. Dai et al. [12] reported significant improvements in balance, walking ability, and quality of life compared to conventional rehabilitation. Similarly, Cugusi et al. [14] showed that aquatic exercise reduces motor impairments and provides slightly to moderately greater benefits than land-based exercise for balance, fear of falling, and health-related quality of life.

Taken together, these data suggest that even moderate-intensity aquatic programs can yield clinically meaningful improvements, situating our intervention along the moderate-to-high dose gradient described in high-intensity exercise studies in PD.

Nonetheless, variability in disease progression necessitates individualized approaches under the supervision of specialized personnel and validated PD-specific functional scales, warranting further studies to define optimal exercise prescriptions [7]. Furthermore, the influence of other lifestyle factors, such as diet, which have implications for neurodegenerative diseases [40,41] but were not examined in this report, deserves further investigation. It is also important to acknowledge that the participant’s male sex may have influenced some of the observed adaptations, particularly those related to muscle strength and body composition. Compared with women, men with PD generally have higher baseline muscle mass, greater absolute strength, and different patterns of fat distribution [42,43]. These differences may partially modulate responsiveness to both resistance and aerobic training. Therefore, the improvements documented in this case may not fully translate to female patients, who often show different sarcopenic trajectories and metabolic responses.

### Limitations of the Study

This case report has potential limitations that restrict the generalizability of its findings. Firstly, the absence of systematic pharmacological and dietary monitoring is a possible source of confounding. Nevertheless, while it may be plausible to regard variations in dopaminergic medication or nutritional intake as potential confounding factors, the patient was in a state of pharmacological stability and maintained the same diet during the period documented by repeated measurements. Secondly, although the participant was clinically monitored, no standardized and validated measures of PD severity were administered during or after the intervention, limiting the capacity to contextualize changes relative to disease progression. Thirdly, body composition was assessed exclusively through anthropometry. While appropriate for monitoring relative within-subject changes, the study lacks complementary objective methods, such as BIA or DXA, to strengthen the accuracy of fat mass and lean mass estimations. Future studies should incorporate these elements to better control potential confounders and support a more robust interpretation of training-related adaptations.

## 5. Conclusions

This case report suggests that a carefully supervised and individually dosed multicomponent adapted physical activity program—combining gym-based training, Nordic walking, and structured swimming—may be safe and potentially beneficial for individuals with mild-to-moderate PD. The improvements observed in body composition, motor performance, and cognitive function underscore the potential value of personalized training pathways in supplementing standard clinical care and boosting patients’ confidence and sense of empowerment.

Moreover, this study enabled us to explore the outcomes from adapted swimming training in a real sporting context, an area in which literature on Parkinson’s disease is lacking. However, these findings must be interpreted with caution, given the single-subject design and the absence of dietary, pharmacological, and PD-specific functional monitoring.

Future research should prioritize longitudinal, multi-participant study designs with repeated measures across the different progression phases, incorporating environmental documentation, standardized warm-up and nutrition protocols, and controlled assessments of medication and nutritional status. The integration of wearable sensors to monitor efficiency, heart rate, and stroke parameters, together with validated clinical scales and objective body-composition techniques, would enhance reproducibility and allow a clearer interpretation of training-induced adaptations, the long-term impact of APA on disease progression, and help identify which patient profiles benefit most from specific exercise modalities.

## Figures and Tables

**Table 1:** Timeline table with milestones.

Timepoint	Test	Adapted Physical Activity
T0 − 4 years	PD Diagnosis	
T0 − 18 months		Start gym-based APA + Nordic walking
T0 − 1 week	First anthropometric measurement	
T0	Baseline: swimming skills assessment, and establishment of individualized training goals	
T0 + 1 week		Start of Adapted swimming training
T1 (8 weeks)	Second motor test (T1)	
T2 (16 weeks)	Third motor test (T2)	
T3 − 1 week	Second anthropometric measurement	
T3 (6 September 2022)	Final motor test (T3)	Participation in the open water Swim 4 Parkinson

**Table 2:** Summary of the Adapted Physical Activity Program.

Modality	Frequency	Intensity (HR)	Time	Adherence (% Completed)	Task Description/Notes
Gym-based APA	1×/week	HR 60–70% max	60 min	95	Mobility, strengthening, and low-intensity aerobic exercises
Nordic Walking	1×/week	HR 65–75% max	50–60 min	100	Outdoor walking with poles; progressive distance based on tolerance
Swimming (pool)	2×/week	HR 60–75% max	40–50 min	90	Technique and endurance training; gradual increase in distance
Swimming (open water)	1×/week (last month)	HR 60–70% max	40–50 min	100	Fully supervised. Focus on orientation, safety, and gradual distance progression according to sea conditions and participant tolerance.

**Table 3:** Anthropometric traits and absolute and percentage changes between pre- and post-swimming intervention (Δ = difference between the second and first measurement, expressed in the unit of measurement of the trait examined; %Δ = percentage difference).

Variables	Pre-Intervention	Post-Intervention	Δ	%Δ
*Somatometric traits*				
Standing height (cm)	158.5	-	-	
Weight (kg)	64.2	60.7	−3.5	−5.5
BMI (kg/m^2^)	25.56	24.16	−1.4	−5.5
WC (cm)	85.4	80.4	−5.0	−5.9
WHtR	0.54	0.51	−0.03	−5.6
Biceps skinfold (mm)	4	3	−1	−25
Triceps skinfold (mm)	9	8	−1	−11.1
Subscapular skinfold (mm)	11	8	−3	−27.3
Suprailiac skinfold (mm)	13	9	−4	−30.8
Sum of skinfolds (mm)	37	28	−9	−24.3
%F	17.1	12.7	−4.4	−25.7
FM (kg)	11.0	7.7	−3.3	−30.0
FFM (kg)	53.2	53.0	−0.2	0.004
FMI (kg/m^2^)	4.38	3.07	−1.31	−29.9
FFMI (kg/m^2^)	21.18	21.10	−0.08	−0.4
MUAC relaxed (cm)	27.2	26.0	−1.2	−4.4
MUAC contracted (cm)	29.9	29.9	0	0
AFI (%)	19.7	18.4	−1.3	−6.6
*Physiometric traits*				
VC (L)	3.92	4.24	+0.32	8.2
FVC (L)	4.27	4.16	−0.11	−2.6
FEV1 (L)	3.40	3.44	+0.04	1.2
Right hand strength (kg)	47.0	48.5	+1.5	3.2
Left hand strength (kg)	45.0	49.5	+4.5	10
DSST (score)	19	23	+4	21.1

Note: BMI = body mass index; WC = waist circumference; WHtR = waist-to-height ratio; %F = fat percentage; FM = fat mass; FFM = fat-free mass; FMI = fat mass index; FFMI = fat-free mass index; MUAC = mid-upper-arm circumference; AFI = arm fat index; VC = vital capacity; FVC = forced vital capacity; FEV1 = forced expiratory volume in one second; DSST = digit symbol substitution test.

## Data Availability

The original data presented in the study are included in the article.

## Supplementary Materials

The following supporting information can be downloaded at: https://www.mdpi.com/article/10.3390/healthcare13243195/s1, Supplementary S1: Adapted Physical Activity program; Supplementary S2: Proposal for a replicable protocol.

## Abbreviations

The following abbreviations are used in this manuscript:

PD	Parkinson disease
APA	adapted physical activity
WC	waist circumference
MUAC	mid-upper-arm circumference
WHtR	waist-to-height ratio
AFI	arm fat index
FM	fat mass
FFM	fat-free mass
FMI	Fat Mass Index
FFMI	Fat-Free Mass Index
BMI	Body Mass Index
VC	Vital capacity
FVC	forced vital capacity
FEV1	forced expiratory volume in one second
DSST	Digit Symbol Substitution Test
SWOLF	swimming golf
MCID	minimal clinically important difference
