# Supplementary material for: Effects of Adapted Physical Activity Programs on Body Composition and Sports Performance in a Patient with Parkinson’s Disease: A Case Report
Source: Healthcare (Basel). 2025 Dec 5;13(24):3195. doi: 10.3390/healthcare13243195 (PMC12732528; doi:10.3390/healthcare13243195)
Supplement: Supplementary file 1 [file healthcare-13-03195-s001.zip › healthcare-3961091-supplementary.pdf]

Supplementary S1

Adapted Physical Activity program (according to FITT principles)

1) Gym-based APA program

Frequency: The sessions were conducted once a week for 22 months

Intensity: Training intensity was progressively adjusted based on the participant's perceived exertion and tolerance, coordination of the limbs, and concentration, aiming for a low to moderate intensity level as aerobic capacity improved.

Time: Each session lasted approximately 60 minutes, structured as follows:

- 10 minutes of warm-up exercises (e.g., joint mobility and gait training)
- 40 minutes of technical exercises that take into account the motor problems of PD patients (e.g., proprioceptive paths, overcoming obstacles, dual task exercises, strength exercises, balance exercises, spatial-temporal orientation exercises with directional changes)
- 10 minutes of relaxation and cool-down

Type: The intervention consisted of APA exercises, progressively including:

- Static exercises in a seated and standing position
- Dynamic exercises using small equipment
- Dynamic exercises in collaboration with other participants
- Dynamic balance exercises with proprioceptive paths using destabilising surfaces (e.g., skinny boards, proprioceptive boards)

2) Nordic walking program

Frequency: The sessions were conducted once a week for 22 months.

Intensity: Training intensity was progressively adjusted based on the participant's perceived exertion and tolerance, coordination of the limbs, and concentration, aiming for a low to moderate intensity level as aerobic capacity improved.

Time: Each session lasted approximately 50 to 60 minutes, structured as follows:

- 7-10 minutes of warm-up exercises (e.g., joint mobility, upper and lower limb strength activation, gait training)
- 40 minutes of Nordic walking with two different styles: parallel 3 technique and alternating technique
- 7-10 minutes of stretching and cool-down.

Type: The intervention consisted of adapted walking exercises, progressively including:

- Parallel 3 technique, as it is simpler in terms of movement coordination
- Alternating technique, which requires a higher degree of coordination

3) Swimming (pool) program

Frequency: The sessions were conducted twice a week for 4 months

Intensity: Training intensity was progressively adjusted based on the participant's perceived exertion and tolerance, coordination of the limbs, and concentration, aiming for a low to moderate intensity level as aerobic capacity improved. Training intensity was monitored using heart rate, which should not exceed 150 beats per minute.

Time: Each session lasted approximately 40 to 50 minutes, structured as follows:

- 7–10 minutes of warm-up exercises (e.g. 100 m at breaststroke 1' rest)
- 25–30 minutes of targeted technical drills (e.g., 100 m (25 m breaststroke with left arm, 25 m breaststroke with right arm, 25 m switch arm every time, 25 m breaststroke) 45'' rest; 100 m (50 m legs breaststroke/arms freestyle, 50 m freestyle) 45'' rest; 200 m breaststroke 2' rest. This training will repeat 3 times.
- 7–10 minutes of cool-down (e.g. walking in warm water (32°C), floating and relaxing with a pool pipe

Type: The intervention consisted of adapted swimming exercises, progressively including:

- Basic stroke technique drills
- Stroke variation exercises (switching between freestyle, backstroke, breaststroke)
- Style transitions (e.g., from one stroke to another, combination of arm movements in freestyle and leg in breaststroke, during laps)
- Controlled speed is important for aerobic training.

4) Swimming (open water) program

Frequency: The sessions were conducted once a week in the month preceding the event

Intensity: Training intensity was progressively adjusted based on the participant's perceived exertion and tolerance, coordination of the limbs, and concentration, aiming for a low to moderate intensity level as aerobic capacity improved. Training intensity was monitored using heart rate, which should not exceed 150 beats per minute.

Time: Each session lasted approximately 40 to 50 minutes, structured as follows:

8–10 min Warm-up

- Gentle swimming from shoreline to breakwater; 2–3 repetitions
- Short pauses allowed if freezing or bradykinesia appear
- Emphasis on progressive increase in mobility and comfort in water

200–300 m Technique

- 10 breaststroke cycles with head underwater
- 10 breaststroke cycles with head up (helps spatial orientation)
- Focus on: large-amplitude movements (counteracting bradykinesia); smooth breathing rhythm; avoiding sudden, abrupt strokes

800-1000 m Main Set

- Continuous, steady-paced breaststroke
- Maintain constant rhythm and stroke length
- Aim for rhythm, wide, relaxed leg movements, stable head position for orientation.

5-7 min Cool-down

- Very light breaststroke back toward shore
- Relaxed breathing with emphasis on long exhalations (supports relaxation of rigidity)

Type:

- Open-water breaststroke (stable, symmetrical, reduces risk of imbalance)
- Technique training for orientation (head-up breaststroke)
- Moderate aerobic continuous swim
- Functional adaptation to waves, currents, and sighting

Supplementary S2

Proposal for a replicable protocol

A proposal for a replicable protocol contains instructions for: a safety checklist, progressions, and criteria for transitioning from pool to open sea.

- **Safety checklist**: We propose a standardized pre-session checklist including environmental assessment (water temperature, currents, visibility), swimmer health status, mandatory safety equipment (wetsuit as needed, brightly coloured cap, safety buoys), communication procedures with lifeguards, and emergency response readiness.

- **Progression model**: A stepwise progression from controlled environments to open-water exposure is suggested. This includes:

1. Pool-based phase: technique stabilization, pace control, and simulated open-water drills (sighting, drafting, turns);

2. Semi-open environment (e.g., sheltered bay): introduction of mild variability (small waves, reduced visibility), supervised by safety personnel;

3. Open-sea phase: gradual increase in distance and duration, exposure to moderate environmental variability, and performance assessments.

- **Criteria for transitioning from pool to open sea**: Swimmers should demonstrate consistent pacing over predetermined distances, stable SWOLF/efficiency metrics, ability to perform sighting without loss of speed or stroke mechanics, and completion of a supervised trial in a semi-open setting without signs of distress.
